# Mutational and transcriptional alterations and clinicopathological factors predict the prognosis of stage I hepatocellular carcinoma: Prediction of stage I HCC prognosis

**DOI:** 10.1186/s12876-022-02496-3

**Published:** 2022-09-24

**Authors:** Zhiqiang Li, Hongqiang Gao, Xiang Zhang, Qiyu Liu, Gang Chen

**Affiliations:** Department of Hepatobiliary Pancreatic Surgery, The First People’s Hospital of Kunming, Kunming, 650032 Yunnan Province China

**Keywords:** Hepatocellular carcinoma, HCC, Mutation, Transcription, Prognosis, Surgery, Radiofrequency ablation

## Abstract

**Background:**

The prognosis of hepatocellular carcinoma (HCC) has been extensively studied. However, the impact on prognosis of stage I HCC has not been well studied at clincopathological, mutational and transcriptional levels.

**Methods:**

Here we first characterized the influencing factors of prognosis of stage I HCC patients by downloading and analyzing the whole-exome somatic mutation data, messenger ribonucleic acid (mRNA) transcription data, along with demographic and clinical information of 163 stage I HCC patients from the TCGA database. The relationship between the influencing factors and HCC prognosis was studied in detail, and a prediction Nomogram model was established. Figures and tables were plotted using the R software.

**Results:**

TP53, CTNNB1, TTN, MUC16 and ALB were the top mutated genes in stage I HCC. A series of co-mutations and mutually exclusive mutations were identified. Twenty-nine genes with significant stratification on prognosis were identified, including highly mutated LRP1B, ARID1A and PTPRQ. Patients with wild type (WT) genes unanimously exhibited significantly better overall survival rate than those with mutants. Patients with the top 10% tumor mutational burden (TMB) exhibited significantly worse prognosis than the rest 90%. Further characterization of transcriptional profile revealed that membrane functions, cell skeleton proteins, ion channels, receptor function and cell cycle were comprehensively altered in stage I HCC. Univariate and multivariate analyses were performed at clinicopathological, mutational and transcriptional levels. The combined analysis revealed sex, race, TMB, neoplasm histologic grade, Child–Pugh grade, MMRN1, OXT and COX6A2 transcription as independent risk factors. These factors were used to establish a Nomogram model to predict the prognosis of individual HCC patients.

**Conclusions:**

The influencing factors of prognosis of stage I HCC have been characterized for the first time at clinicopathological, mutational and transcriptional levels. A Nomogram model has been established to predict the prognosis. Further validation is needed to confirm the effectiveness and reliability of the model.

**Supplementary Information:**

The online version contains supplementary material available at 10.1186/s12876-022-02496-3.

## Introduction

HCC ranked the 5th in morbidity and 2nd in mortality in China [1]. It was reported that the HCC patients in China account for nearly half of the HCC new cases and death worldwide and therefore became a big component in burden of disease in China [2]. The 5-year survival rate of HCC was reported to be 14.6% in China and 19.6% in the US, much lower than other cancers [2, 3]. This was partially due to the lack of screening and early detection of HCC when the tumor is still resectable and can be cured. With the rapid development of early screening methods, more and more HCC patients were identified at early stages, which may potentially improve the survival rate and life quality of patients. Hepatitis B infection and subsequent liver cirrhosis is the main cause of HCC in China while alcoholic liver cirrhosis is the main cause of HCC in Western countries [4].

Early-stage HCC is generally defined as stage I HCC by tumor, node, metastasis (TNM) staging system or Barcelona clinic liver cancer (BCLC) 0 or A by the BCLC staging system [5, 6]. These patients can potentially be cured by radical surgery or radiofrequency ablation (RFA), and therefore exhibite much better prognosis than those with late-stage HCC. The influencing factors of HCC prognosis has been extensive studied. These include a series of clinicopathological factors and many biomarkers at mutational, transcriptional and expression levels [7]. Several aberrant pathways, including Janus kinase/signal transducer and activator of transcription (JAK/STAT), phosphotylinosital 3 kinase/protein kinase B-mammalian target of rapamycin (PI3K/AKT-mTOR), mitogen-activated protein kinase (MAPK), transforming growth factor-β (TGF-β) and wingless (Wnt) pathways, has been identified in previous reports, suggesting comprehensive abnormalities and huge heterogeneity of HCC [7–9]. Although the treatment strategies and prognosis of late-stage HCC have been widely studied, the prognosis of early-stage HCC and its influencing factors have not been well defined, partially because these patients generally had longer survival and it took a long time for the endpoints of prognosis study to be reached.

Here in this study, we focused on the influencing factors of prognosis of Stage I HCC patients (AJCC guideline), and aimed to establish a model to predict the prognosis of these patients. We planned to examine the clinicopathological factors, mutational status and transcriptional profile by downloading the corresponding data of stage I HCC patients from The Cancer Genome Atlas (TCGA) database. Analyses of relationship between prognosis and these factors were performed and the results were used in establishing a prediction model. We believe that the model will be useful for predicting the long-term survival of stage I HCC patients and providing an expectation of prognosis before any therapy. Further validation of the model may be needed to confirm its effectiveness and reliability.

## Methods and materials

The whole-exome somatic mutation data, mRNA transcription data, along with demographic and clinical information of 163 stage I HCC patients were downloaded from The Cancer Genome Atlas (TCGA) database (https://portal.gdc.cancer.gov/). Here HCC staging is defined by the American Joint Committee on Cancer (AJCC) guideline, in which stage I HCC includes stage IA and IB, corresponding to T1aN0M0 and T1bN0M0 by TNM staging. Data files in Mutation Annotation Format (MAF) format were obtained using the “TCGAbiolinks” package of R software (https://www.rstudio.com/). Mutation profile and TMB were analyzed using the “maftools” of R software. The read counts of the transcription data were obtained by HTSeq-count software, and the differential transcription was analyzed by the “edgeR” package of the R software. Patient demographic and clinical information is summarized in Table 1.

**Table 1:** Summary of clinicopathological factors of patients invovled in this study

Factors	Categories	Number (%)
Sex (%)	Female	47 (28.8)
	Male	116 (71.2)
Age (mean (SD))		60.31 (12.11)
Race (%)	Asian	78 (47.8)
	Black or African American	7 (4.3)
	Not reported	6 (3.7)
	White	72 (44.2)
TMB (mean (SD))		1.52 (1.03)
Neoplasm histologic grade (%)	G1	25 (15.3)
	G2	75 (46.0)
	G3	52 (31.9)
	G4	11 (6.7)
Adjacent hepatic tissue inflammation (%)	None	55 (33.7)
	Mild or severe	56 (34.4)
	Not reported	52 (31.9)
Child Pugh classification grade (%)	A	118 (72.4)
	B or C	10 (6.1)
	Not reported	35 (21.5)
Fibrosis Ishak score (%)	0: No fibrosis	28 (17.2)
	1, 2: Portal fibrosis	18 (11.0)
	3, 4: Fibrous speta	13 (8.0)
	5: Nodular formation and incomplete cirrhosis	1 (0.6)
	6: Established cirrhosis	42 (25.8)
	Not reported	61 (37.4)
Creatinine (%)	Normal	114 (69.9)
	High	8 (4.9)
	Low	26 (16.0)
	Not reported	15 (9.2)
Platelet (%)	Normal	109 (66.9)
	High	4 (2.5)
	Low	39 (23.9)
	Not reported	11 (6.7)
Albumin (%)	Normal	114 (7.0)
	High	1 (0.6)
	Low	33 (20.2)
	Not reported	15 (9.2)

TMB, tumor mutational burden; G, grade; SD, standard deviation

All patients were divided into mutation group (Mut) and wild type group (WT) in mutational status analysis. Patients were also divided into high and low transcription in transcriptional level analysis. Kruskal–Wallis test was performed by R software to compare the difference among groups with different mutational or transcriptional status. For the differentially transcripted genes revealed by ribonucleic acid sequencing (RNA-seq), variable selection was carried out using the "GLmnet" LASSO (Least Absolute Shrinkage and Selection Operator) regression algorithm in R software. The ‘maftools’ package of the R software was used for plotting the mutational landscape and characteristics. The ‘pheatmap’, ‘ggplot2’ and ‘clusterProfiler’ packages of the R software were used for plotting heatmap, volcano plot and the results for gene ontology (GO), Kyoto encyclopedia of genes and genomes (KEGG) and Reactome enrichment analysis, respectively. The relationship between patient prognosis and clinicopathological factors, mutational status or mRNA levels was analyzed by univariate and multivariate analyses. Kaplan–Meier analysis and log-rank test were performed by R software to investigate the potential stratification of mutations or transcription on patient overall survival. Survival curves were plotted using the ‘survival’ and ‘survminer’ package of the R software. The Nomogram model was plotted with the ‘rms’ package of the R software. *P* values were adjusted by Benjamini & Hochberg (BH) method. *P < 0.05; **P < 0.01; ***P < 0.001.

## Results

### The mutational landscape and transcriptional profile of stage I HCC

**Fig. 1:**
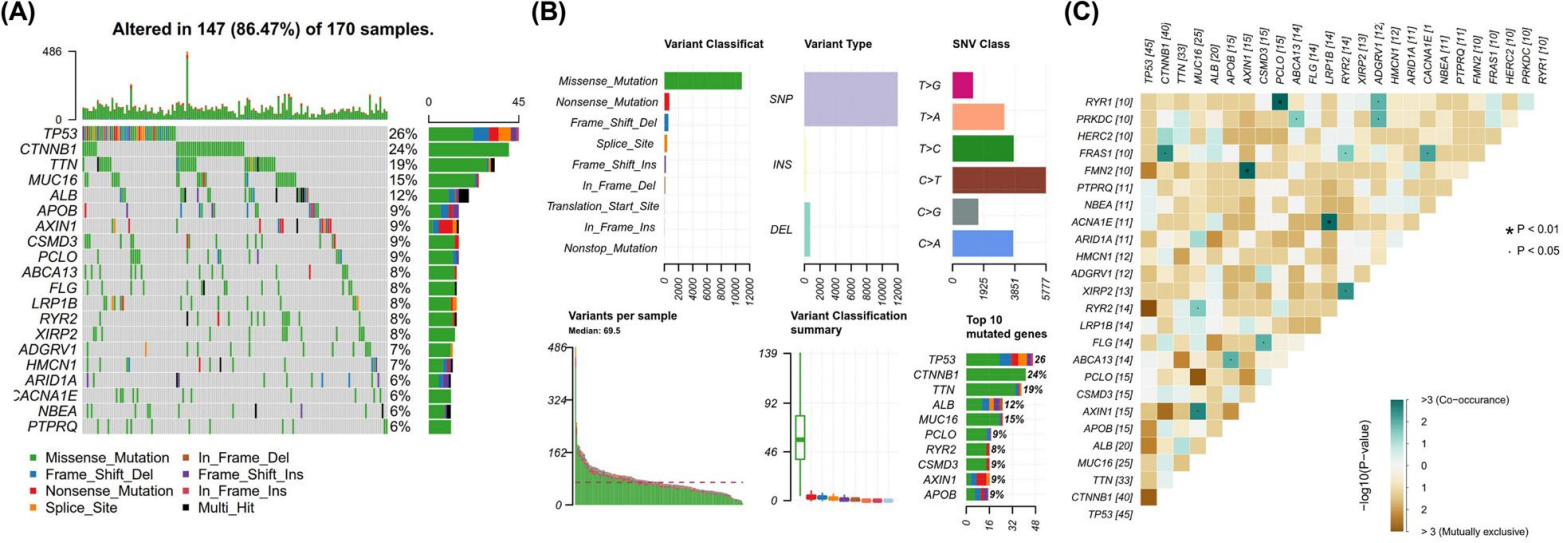
Mutational landscape, characteristics and mutational correlation in stage I hepatocellular carcinoma (HCC). **A** mutational landscape of 163 stage I HCC patients. The mutational status of the top 20 mutated genes is shown as indicated; **B** mutational characteristics of stage I HCC, including variant classification, type, rank and mutational burden. **C** Status of co-mutations and mutually exclusive mutations. The status and co-mutations or mutually exclusive mutations are indicated by colors and *P* values. SNV, single nucleotide variant; Del, deletion; Ins, insertion

In order to investigate the mutational status of stage I HCC, the mutation data of 163 stage I HCC patients were downloaded and analyzed, and the mutational landscape was established by plotting the oncoplot (Fig. 1A). It showed that TP53, CTNNB1, TTN, MUC16 and ALB were the top mutated genes, with mutational frequency higher than 10%. Analysis on mutational characteristics showed that single nucleotide variant (SNV) mutation was the predominant variant type, and missense mutation was the predominant type in SNV mutations (Fig. 1B). Huge variation in the number of variants per sample was observed, with a median of 69.5 variants per sample. TP53, CTNNB1, TTN, ALB and MUC16 were genes with highest number of mutations (Fig. 1B). A series of co-mutations and mutually exclusive mutations were identified (Fig. 1C). For example, PCLO was co-mutated with RYR1 (*P* < 0.01), AXIN1 was co-mutated with FMN2 (*P* < 0.01), and LRP1B was co-mutated with ACNA1E (*P* < 0.01), while TP53 exhibited a trend of mutually exclusive mutated with RYR2 and CTNNB1.

**Fig. 2:**
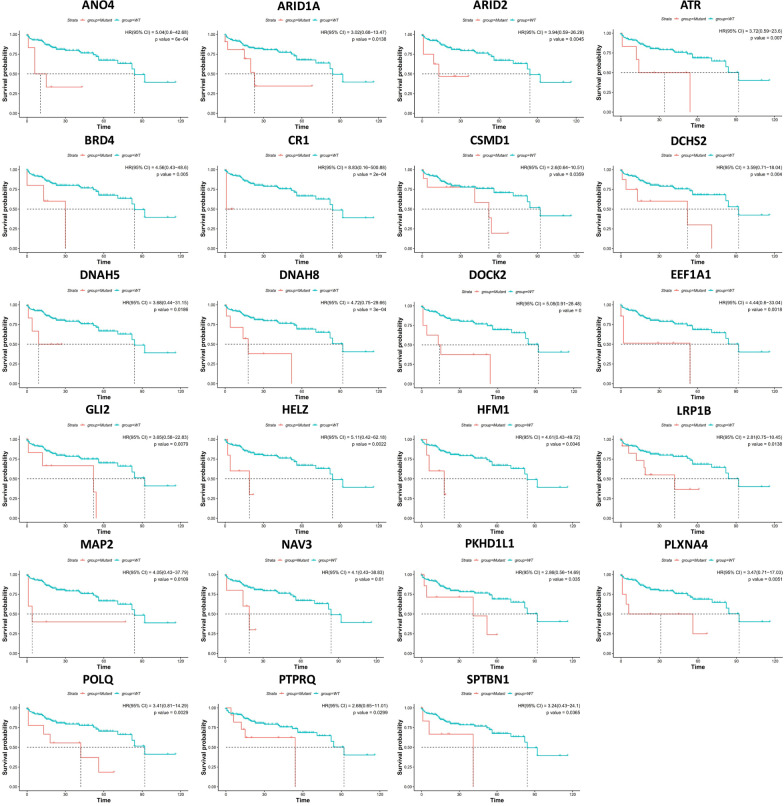
Genes with significant stratification of prognosis by mutational status. The top 200 mutated genes in stage I hepatocellular carcinoma (HCC) were examined and those with significant stratification of prognosis are shown here. The mutational status is indicated by different colors and *P* values and hazard ratio (HR) values are shown as indicated. The unit for time is months. HR, hazard ratio; WT, wild type; CI, confidence interval

The stratification by mutational status on stage I HCC patient prognosis was further studied. The stratification of patient prognosis by the mutational status of the top 200 mutated genes was examined, and 29 genes with significant stratification were identified (Fig. 2). It can be seen that some top mutated genes were among the 29 genes, including LRP1B, ARID1A and PTPRQ, although a large majority of genes were those with low mutational frequency. It was also interesting to find that patients with WT genes exhibited significantly better survival rate than those with mutant genes in all 29 genes, suggesting a common trend in stratification by mutational status.

**Fig. 3:**
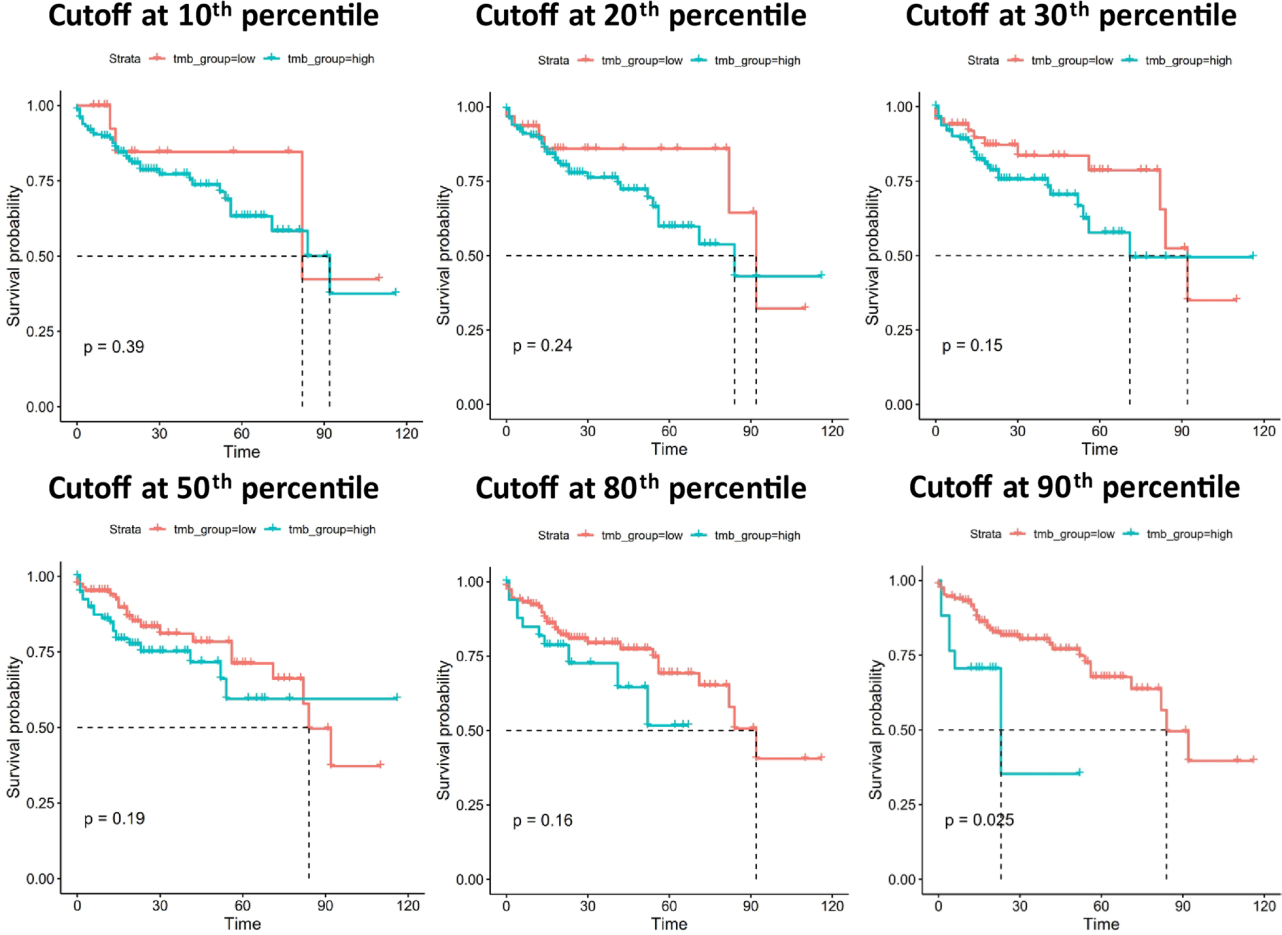
The stratification of patient prognosis by tumor mutational burden (TMB). The stratification status by a series of thresholds at different percentile is shown here. Only cutoff at 90th percentile was found to significantly stratify the patient prognosis, although all stratification showed a trend that low TMB group exhibited a better prognosis. The unit for time is months

TMB is a well-known stratification for many types of cancers, including HCC [10, 11]. Here we examined the potential stratification by TMB in stage I HCC. Different TMB thresholds were examined by selecting a series of cutoff values at different percentile (Fig. 3). Our analysis showed that patients in the low TMB group all exhibited a trend of better survival rate than those in the high TMB group, although statistically significant stratification was only observed at 90th percentile (*P* = 0.025). This observation suggested that patients with TMB at the top 10% exhibited significantly worse prognosis than the rest 90%.

**Fig. 4:**
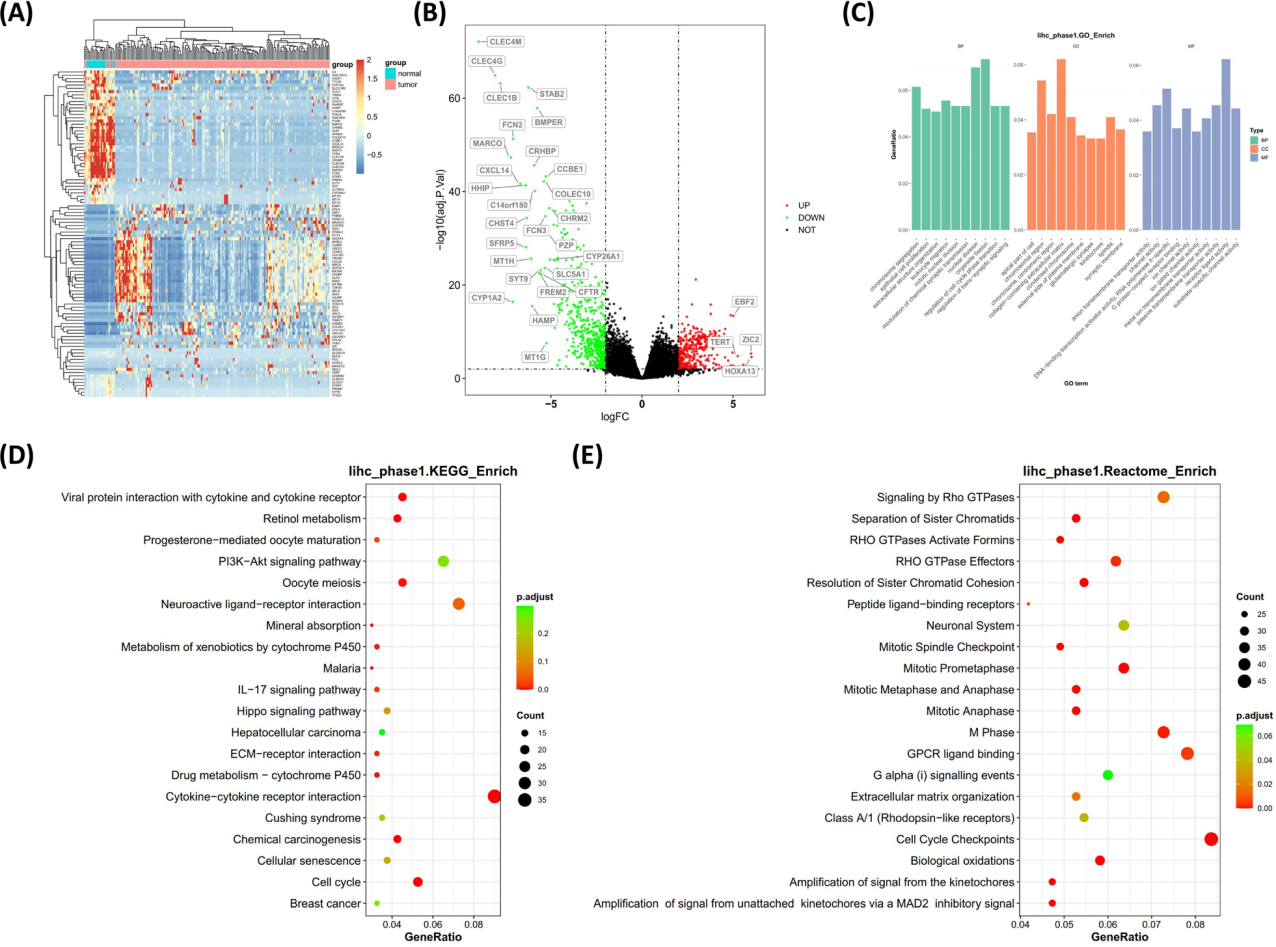
Profile of transcriptional alterations of stage I hepatocellular carcinoma (HCC). **A** The heatmap shows the transcriptional status of the top 100 differentially transcripted genes. Blue bars represent normal tissues while pink bars represent cancer tissues. **B** Volcano plot shows the significantly up-regulated (red) or down-regulated (green) genes, with the names of the most significant ones labeled. **C**–**E** The results for GO (**C**), KEGG (**D**) and Reactome (**E**) enrichment analyses. The significantly altered functions or pathways are shown as indicated. GO, gene ontology; KEGG, Kyoto encyclopedia of genes and genomes; BP, biological process; CC, cellular compartment; MF, molecular function; FC, fold change; p.adjust, adjusted *p* value

The transcriptional profile of stage I HCC was next investigated by examining the alterations at mRNA level. Heatmap in Fig. 4A shows the top 100 significantly altered genes in HCC tissues compared with normal tissues. Substantial up-regulation or down-regulation can be observed in HCC tissues. The profile of up- or down-regulated transcription was shown by the volcano plot in Fig. 4B, in which significant up-regulation (red dots) or down-regulation (green dots) can be defined by cutoff values at |logFC| = 2 and − log10 (adj.*P*.Val) = 2 (adjusted *P* < 0.01). The most up-regulated or down-regulated genes were labeled in the Fig. 4B, including the well-known telomerase reverse transcriptase gene TERT, which appeared to be significantly up-regulated. The altered physiological functions and signaling pathways were further investigated by performing the enrichment analyses. GO enrichment revealed that membrane functions, cellular skeleton proteins and ion channels were mostly involved in stage I HCC aberrancies (Fig. 4C). KEGG enrichment (Fig. 4D) and Reactome enrichment (Fig. 4E) suggested that receptor function and cell cycle were comprehensively affected in stage I HCC.

### The influencing factors and predictive model for the prognosis of stage I HCC

To identify the influencing factors and establish a model for predicting the prognosis of stage I HCC patients, we performed univariate and multivariate analyses in three aspects, including the clinicopathological factors (Table 2), the mutational status (Table 3) and the transcriptional status (Table 4). Univariate analysis on clinicopathological factors revealed age, sex, race and TMB as the significant risk factors, while further multivariate analysis revealed neoplasm histologic grade, Child–Pugh classification grade, BMI, age and race as the independent risk factors (Table 2). Subsequent univariate analysis on top 200 mutated genes revealed that ARID1A, LRP1B and PTPRQ were significantly related to the prognosis of stage I HCC patients, while these three genes were also significant in multivariate analysis (Table 3). Univariate analysis on transcriptional status identified 79 genes significantly related to prognosis (Additional file 1: Table S1), and multivariate analysis confirmed 6 genes with significant influence on prognosis, including ABCB5, XG, FAM9B, LYVE1, COX6A2 and OXT (Table 4).

**Table 2:** Univariate and multivariate analyses of clinicopathological factors

	Univariate		Multivariate	
	HR (95% CI)	*P* value	HR (95% CI)	*P* value
*Neoplasm histologic grade*				
G1	Reference		Reference	
G2	1.07 (0.42–2.70)	0.886	1.38 (0.29–6.50)	0.681
G3	0.84 (0.31–2.26)	0.723	2.74 (0.49–15.33)	0.252
G4	2.08 (0.57–7.54)	0.266	10.78 (1.30–89.59)	**0.028**
*Child Pugh classification grade*				
A	Reference		Reference	
B or C	1.85 (0.64–5.36)	0.255	10.28 (1.82–57.98)	**0.008**
*BMI*				
Normal	Reference		Reference	
High	1.36 (0.70–2.67)	0.363	0.30 (0.10–0.89)	**0.029**
Low	0.00 (0.00-Inf)	0.997	0.00 (0.00-Inf)	0.998
*Age*				
Age	1.04 (1.02–1.07)	**0.003**	1.08 (1.02–1.14)	**0.007**
*Sex*				
Female	Reference		Reference	
Male	0.49 (0.27–0.92)	**0.025**	0.76 (0.24–2.38)	0.639
*Race*				
Asia	Reference		Reference	
Black or African American	5.95 (1.62–21.88)	**0.007**	25.53 (3.97–164.23)	**0.001**
White	3.05 (1.47–6.33)	**0.003**	3.86 (0.86–17.35)	0.078
*TMB*				
Low	Reference		Reference	
High	2.69 (1.10–6.58)	**0.030**	1.97 (0.30–12.88)	0.480
*Fibrosis Ishak score*				
0: No fibrosis	Reference		Reference	
1, 2: Portal fibrosis	1.46 (0.41–5.17)	0.556	1.18 (0.15–9.43)	0.877
3, 4: Fibrous speta	1.49 (0.43–5.14)	0.530	1.86 (0.44–7.79)	0.397
5, 6: Incomplete or established cirrhosis	0.79 (0.30–2.07)	0.634	1.89 (0.51–7.08)	0.344

*P* < 0.05 for bold* P* values

HR, hazard ratio; CI, confidence interval; G, grade; BMI, body mass index; TMB, tumor mutational burden

**Table 3:** Univariate and multivariate analyses of the top 20 mutated genes

	Univariate		Multivariate	
	HR (95% CI)	*P* value	HR (95% CI)	*P* value
*ARID1A*				
WT	Reference			
Mut	3.02 (0.68–13.47)	0.0138	4.19 (1.57–11.19)	0.0042
*LRP1B*				
WT	Reference			
Mut	2.81 (0.75–10.45)	0.0138	3.80 (1.53–9.43)	0.0040
*PTPRQ*				
WT	Reference			
Mut	2.68 (0.65–11.01)	0.0299	3.75 (1.40–10.03)	0.0086

HR, hazard ratio; CI, confidence interval; WT, wild type; Mut, mutant

**Table 4:** Multivariate analysis of singificantly differential transcripted genes

	Multivariate	
	HR (95% CI)	*P* value
MMRN1	1.15 (0.94–1.40)	0.179
ABCB5	1.24 (1.09–1.40)	**0.001**
XG	1.28 (1.09–1.51)	**0.003**
FAM9B	1.24 (1.06–1.44)	**0.006**
LYVE1	1.36 (1.07–1.72)	**0.013**
COX6A2	1.18 (1.05–1.32)	**0.006**
OXT	0.71 (0.61–0.83)	**0.000**

*P* < 0.05 for bold* P* values

HR, hazard ratio; CI, confidence interval

**Fig. 5:**
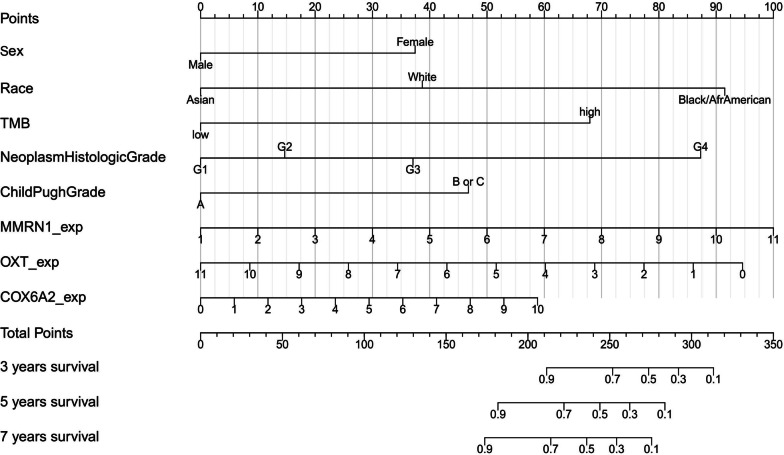
The Nomogram model for predicting the prognosis of stage I hepatocellular carcinoma (HCC) patients. A series of clinicopathological factors, including sex, race, neoplasm histological grade, Child–Pugh grade, and the mutational status, represented by tumor mutational burden (TMB), and transcriptional levels of three genes, including MMRN1, OXT and COX6A2, were used to establish the model. TMB, tumor mutational burden; exp, expression; G, grade; Afr, African

All significant clinicopathological factors, mutated genes and differentially transcripted genes were included in a final multivariate analysis to identify the significant influencing factors of prognosis when factors from these three levels were analyzed together. The results in Table 5 show that sex, race, TMB, neoplasm histologic grade, Child–Pugh grade, MMRN1, OXT and COX6A2 transcription (or expression, exp) were independent risk factors for the prognosis of stage I HCC patients. These factors were used to establish a Nomogram model to predict the prognosis of individual HCC patients (Fig. 5). Based on the available overall survival data from the TCGA database, the probability for 3-year, 5-year and 7-year survival can be quantified by calculating the total points from the score of each factor. Further validation of the model is needed to confirm its effectiveness and reliability.

**Table 5:** Multivariate analysis of clinicopathological, mutational and transcriptional factors on patient prognosis

	Multivariate	
	HR (95% CI)	*P* value
*Sex*		
Female	Reference	
Male	0.32 (0.11–0.96)	**0.041**
*Race*		
Asia	Reference	
Black or African American	15.92 (3.10–81.68)	**0.001**
White	3.22 (1.09–9.53)	**0.034**
*TMB*		
Low	Reference	
High	7.81 (2.24–27.24)	**0.001**
*Neoplasm histologic grade*		
G1	Reference	
G2	1.56 (0.37–6.50)	0.542
G3	3.07 (0.63–15.03)	0.166
G4	14.01 (1.98–99.03)	**0.008**
*Child Pugh grade B or C*		
A	Reference	
B or C	4.11 (1.11–15.21)	**0.034**
*MMRN1_exp*	1.35 (1.00–1.83)	**0.050**
*OXT_exp*	0.77 (0.62–0.96)	**0.019**
*COX6A2_exp*	1.19 (1.03–1.39)	**0.023**

*P* < 0.05 for bold* P* values

HR, hazard ratio; CI, confidence interval; TMB, tumor mutational burden; G, grade; exp, expression

## Discussion

In this study, we identified the prognostic factors for stage I HCC by examining the clincopathological features, the mutational characteristics and the transcriptional profile, and combined the significant factors from the three aspects to establish a model and predicted the long-term prognosis of this specific group of patients. We identified a series of clinicopathological factors, including sex, race, neoplasm histologic grade and Child–Pugh grade as the independent risk factors. We also identified mutational status, represented by TMB, as one independent risk factor. Transcriptional alterations of three genes, including MMRN1, OXT and COX6A2, were also identified as independent risk factors. The Nomogram model was established based on the combination of these independent risk factors, which we believe will be a reasonable tool for predicting the prognosis of stage I HCC patients.

The prognosis of HCC was not only affected by tumor characteristics, but also by liver function, which was a key prognostic factor for survival. Apart from liver function, portal hypertension and other complications were also important factors affecting survival, as most HCC patients had underlying chronic liver disease or cirrhosis [12–14]. Therefore, clinicopathological factors played an important role in predicting the prognosis of HCC. TNM and BCLC staging systems provide a theoretical basis for clinical decision making [15, 16]. TNM staging mainly considers tumor size, vascular, nerve, lymph node invasion and local and distal metastasis. BCLC staging involves different prognostic, clinical, and tumor burden variables. Total bilirubin, portal hypertension status, liver function, other complications, and the Eastern Cooperative Oncology Group (ECOG) status are associated with prognosis.

However, novel biomarkers at mutational, epigenetic and transcriptional levels have not been included in the current assessment of HCC prognosis. At present, other staging systems mainly consider the influence of alpha fetal protein (AFP) on HCC staging and prognosis, such as the Cancer of the Liver Italian Program (CLIP) [17], French GRETCH staging [18] and Chinese University Prognosis Index (CUPI) [19]. Staging system with biomarkers alone, such as the BALAD score, has also been established. BALAD score included two biochemical variables (serum bilirubin and albumin) and three biomarkers (AFP > 400 ng/mL, AFP-L3 > 15%, and Des-γ-carboxy-prothrombin (DCP) > 100 mAU/mL) [20], whose combination was associated with a poor prognosis. However, this score has not been widely used in daily practice because it does not include any imaging tumor features or clinically relevant data.

The relationship between AFP and prognosis has been extensively studied. The increase of AFP level was associated with low survival rate and high tumor recurrence rate in early-stage patients [21–24] and poor prognosis in advanced HCC patients [25, 26]. In intermediate stage patients, AFP was associated with tumor progression in patients awaiting liver transplantation or receiving local tumor therapy to reduce the tumor burden [27, 28]. Serum AFP values over 400 ng/mL were associated with higher tumor progression rate and lower response rate after trans-arterial chemoembolization (TACE) [29, 30]. DCP or protein induced by vitamin K absence or antagonist-II (PIVKA-II) was also considered as prognostic markers for HCC. It was shown to be associated with larger tumors, poor differentiation and vascular invasion [31, 32]. Compared with AFP, it was specifically associated with vascular invasion [33]. It was also shown that DCP serum levels increased after hypoxia, and was proposed as a predictive biomarker after anti-angiogenic therapy [34]. DCP was associated with lower survival and higher risk of HCC recurrence after hepatectomy [35, 36].

In this study, we identified mutational status of three genes, including ARID1A, LRP1B and PTPRQ, and TMB as the independent risk factors. It was interesting to find that patients with WT genes exhibited better prognosis than those with mutant counterparts, which was consistent with the trend that patient with low TMB exhibited better survival than those with high TMB. These results indicated that patients with less mutations appeared to have better survival than those with more mutations in stage I HCC, which was contrary to the findings in early-stage lung cancer, in which patients with high TMB exhibited better prognosis [37, 38]. This observation suggested that different cancers varied greatly in their correlation between TMB and prognosis. In our final Nomogram model, ARID1A, LRP1B and PTPRQ mutational status was not included. This was because their mutational status was positively correlated with TMB, which appeared to be a more representative and comprehensive marker for prognosis prediction.

HCC is highly heterogeneous with a large number of mutations. The most common ones were TERT, TP53 and CTNNB1 [39–41]. TERT promoter mutations were most common in 60% of HCC cases, while TP53 and CTNNB1 were present in about 30%. Two different categories of HCC have been proposed based on genomic profiles and their correlation with phenotypic profiles. The proliferative type has been associated with poor prognosis, TP53 mutations, and genetic characteristics of chromosomal instability. This type of proliferation is associated with hepatitis B virus (HBV) infection, poor cell differentiation, high AFP and poor survival. In contrast, the non-proliferative type was associated with CTNNB1 mutations, immune rejection, hepatitis C virus (HCV) and alcoholic liver disease, lower tumor grade, lower frequency of vascular invasion, and better prognosis [39–42]. DNA methylation, by contrast, may be more specific than mutations. DNA methylation is an important mechanism of epigenetic regulation of gene expression and has been reported in HCC [43, 44]. The tissue-specific methylation patterns are potentially biomarkers for early HCC diagnosis or prognosis, whether in liquid biopsies or tumor samples.

Several reports supported the role of genetic alterations in predicting the prognosis of HCC patients. This included tumor mutational burden [45, 46], TP53 [47, 48] and CTNNB1 [49, 50] mutations. Studies reported a negative correlation between TMB and the prognosis of HCC patients (higher TMB correlated with poorer prognosis) [45, 46]. It was also found that the TP53 mutation rate in the high-risk group was significantly higher than those in the low-risk group, and TP53 249Ser mutation may be a high-risk factor of HBV-related HCC recurrence in the short term [47, 48]. Furthermore, a metabolic prognostic model associated with CTNNB1 mutations could be implemented for determining the prognoses of individual patients. CTNNB1 mutations was also found to be potential biomarkers for HCC immunotherapy patients, as it identified patients less likely to benefit from immune checkpoint inhibitors [49, 50].

The malignant transformation in HCC may happen in two ways [51]. Most HCC undergoes canonical pathway: hepatic cirrhosis-low grade dysplasia-high grade dysplasia-early HCC-advanced HCC. These HCC cases show aberrations involving a series of high-frequency mutations (TERT promoter, TP53, CTNNB1, ARID1A, etc.). Patients in this group generally have long HBV infection and cirrhosis history. Effective control of HBV infection and early intervention on precancerous or early cancerous lesions may provide good prognosis. In contrast, a small portion of HCC may derive from normal HCC without the above canonical process. This includes HBV DNA insertional mutagenesis or direct effect of viral oncoprotein, specific DNA mutagenesis due to toxin (such as Aflatoxin B1) and female oral contraception (liver adenoma malignant transformation). Insertional mutagenesis in TERT, CCNA2, CCNE1, TNFSF10 and MLL4 may be observed [51]. The sequence of events differs in hepatocellular adenoma compared with cirrhosis. In normal hepatocytes, CTNNB1 activating mutation occurs first and is associated with monoclonal benign proliferation at risk of transformation, while in cirrhotic hepatocytes, TERT promoter mutation occurs earlier [51]. These patients may show earlier HCC onset in lifetime and advanced HCC when first diagnosed, and therefore may have worse prognosis than those with canonical pathway.

At present, liquid biopsy has become a new research hotspot. Different forms of liquid biopsy included circulating tumor cells, circulating tumor deoxyribonucleic acid (ctDNA), microRNA, and extracellular vesicles. However, the liquid biopsy method is facing some challenges [52–54]. First, the number of circulating tumor cells is a challenge, especially in the early stages. Second, ctDNA makes up less than 1% of the total amount of free DNA circulating and may not reflect tumor-specific DNA. Instead, it may reflect necrotic or apoptotic tumor cells, and may not even be cells from tumor tissue. In addition, ctDNA mutations may not be specific to HCC in the context of cirrhosis. However, liquid biopsy is not only being studied for the early detection of liver cancer, but also as a prognostic tool. Copy number variation, gene integrity, mutations and DNA methylation changes have potential applications in the diagnosis, treatment and prognosis assessment of HCC [44].

The prognosis of stage I HCC patients may be influenced by different therapeutic strategies. The therapeutic strategies for stage I HCC include surgical resection and RFA. Studies have shown that the long-term prognosis of HCC was different between the two groups. In a meta-analysis [55], the investigators included five trials with a total of 742 patients. Analysis showed that 1-year and 3-year overall survival was similar for RFA and surgical resection, while the 5-year overall survival decreased in RFA compared with surgical resection. The total recurrence rate was also significantly higher in the RFA group than the surgical resection group, while the length of hospital stay was significantly shorter with RFA [55]. In another study [56], 2865 patients with liver surgical resection and 2764 patients with local ablation (RFA, microwave ablation (MWA), RFA + TACE) were included in a meta-analysis. Although there was no significant difference in OS between surgical resection and RFA group, the 5-year recurrence free survival (RFS) of the surgical resection group was significantly better than RFA group. The RFA group showed significantly higher recurrence rate than surgical resection group. In addition, The OS and RFS of surgical resection group were better than those of MWA or RFA + TACE group. It appeared from the above studies that surgical resection was superior to RFA in terms of RFS and local recurrence rate and therefore may exhibit a better prognosis [56]. The TCGA database does not provide the information on therapeutic response assessment, therefore the comparison of prognosis of stage I patients by therapeutic strategies was not possible in this study with current available data.

This study had some limitations. First, the Nomogram model needs to be validated in retrospective or prospective cohort to ensure its effectiveness and reliability. Future optimization of the model may be required to facilitate its use in the real world. Secondly, therapeutic strategy appeared to be an influencing factor for patient prognosis, and detailed stratification of population may be needed when using the model in clinical practice. Thirdly, the current model requires NGS sequencing on tissue mutations and transcription, which may hinder its use due to the costs and availability of tissue samples. Future tests and model optimization based on blood may be more feasible for easy and quick assessment of patient prognosis.

## Conclusions

The prognostic factors of stage I HCC have been investigated at clinicopathological, mutational and transcriptional levels. Sex, race, TMB, neoplasm histologic grade, Child–Pugh grade, MMRN1, OXT and COX6A2 transcription have been identified as independent risk factors. A prognostic model has been established with these significant prognostic factors, while further validation is needed to confirm its effectiveness.

## Supplementary Information

**Additional file 1: Table S1**. Univariate analysis of transcriptional status revealed significant genes related to the prognosis of Stage I HCC patients. Description of data: A list of main parameters from the univariate analysis.

**Additional file 2: Table S2**. The patient identifier for all subjects involved in this study. Description of data: A list of submitter id of all 163 patients.

## Data Availability

The datasets generated and/or analyzed during the current study are available from the corresponding author upon reasonable request. The patient identifiers (submitter id) of all subjects involved in this study from the TCGA database are listed in Additional file 2: Table S2.

## Abbreviations

AFP: Alpha fetal protein
BCLC: Barcelona clinic liver cancer
BH: Benjamini & Hochberg
ctDNA: Circulating tumor deoxyribonucleic acid
DCP: Des-γ-carboxy-prothrombin
GO: Gene ontology
HBV: Hepatitis B virus
HCC: Hepatocellular carcinoma
HCV: Hepatitis C virus
JAK/STAT: Janus Kinase/Signal Transducer and Activator of Transcription
KEGG: Kyoto Encyclopedia of Genes and Genomes
LASSO: Least absolute shrinkage and selection operator
MAF: Mutation annotation format
MAPK: Mitogen-activated protein kinase
mRNA: Messenger ribonucleic acid
MWA: Microwave ablation
PI3K/AKI-mTOR: Phosphotylinosital 3 kinase/protein kinase B-mammalian target of rapamycin
PIVCA-II: Protein induced by vitamin K absence or antagonist-II
RFA: Radiofrequency ablation
RFS: Recurrence-free survival
RNA-seq: Ribonucleic acid sequencing
SNV: Single nucleotide variant
TACE: Trans-arterial chemoembolization
TCGA: The Cancer Genome Atlas
TGF-β: Transforming growth factor-β
TMB: Tumor mutational burden
TNM: Tumor, node, metastasis
Wnt: Wingless
WT: Wild type
